# Self-supervised learning of wrist-worn daily living accelerometer data improves the automated detection of gait in older adults

**DOI:** 10.1038/s41598-024-71491-3

**Published:** 2024-09-06

**Authors:** Yonatan E. Brand, Felix Kluge, Luca Palmerini, Anisoara Paraschiv-Ionescu, Clemens Becker, Andrea Cereatti, Walter Maetzler, Basil Sharrack, Beatrix Vereijken, Alison J. Yarnall, Lynn Rochester, Silvia Del Din, Arne Muller, Aron S. Buchman, Jeffrey M. Hausdorff, Or Perlman

**Affiliations:** 1 Department of Biomedical Engineering, Tel Aviv University, Tel Aviv, Israel; 2 Center for the Study of Movement, Cognition and Mobility, Neurological Institute, Tel Aviv Sourasky Medical Center, Tel Aviv, Israel; 3 Biomedical Research, Novartis Pharma AG, Basel, Switzerland; 4 Department of Electrical, Electronic and Information Engineering Guglielmo Marconi, University of Bologna, Bologna, Italy; 5 Health Sciences and Technologies-Interdepartmental Center for Industrial Research (CIRI-SDV), University of Bologna, Bologna, Italy; 6 Laboratory of Movement Analysis and Measurement, Ecole Polytechnique Federale de Lausanne, Lausanne, Switzerland; 7 Robert Bosch Gesellschaft für Medizinische Forschung, Stuttgart, Germany; 8 Unit Digitale Geriatrie, Universitätsklinikum Heidelberg, Heidelberg, Germany; 9 Department of Electronics and Telecommunications, Politecnico di Torino, Turin, Italy; 10 Department of Neurology, University Medical Center Schleswig-Holstein, Campus Kiel, Kiel, Germany; 11 Department of Neuroscience and Sheffield NIHR Translational Neuroscience BRC, Sheffield Teaching Hospitals NHS Foundation Trust, Sheffield, UK; 12 Department of Neuromedicine and Movement Science, Norwegian University of Science and Technology, Trondheim, Norway; 13 Translational and Clinical Research Institute, Faculty of Medical Sciences, Newcastle University, Newcastle Upon Tyne, UK; 14 The Newcastle Upon Tyne Hospitals NHS Foundation Trust, Newcastle Upon Tyne, UK; 15 National Institute for Health and Care Research (NIHR) Newcastle Biomedical Research Centre (BRC), Newcastle University, The Newcastle Upon Tyne Hospitals NHS Foundation Trust, Newcastle Upon Tyne, UK; 16 Department of Neurological Sciences, Rush Alzheimer’s Disease Center, Rush University Medical Center, Chicago, IL USA; 17 Department of Physical Therapy, Faculty of Medical and Health Sciences, Tel Aviv University, Tel Aviv, Israel; 18 Sagol School of Neuroscience, Tel Aviv University, Tel Aviv, Israel; 19 Rush Alzheimer’s Disease Center and Department of Orthopedic Surgery , Rush University, Chicago, IL USA

**Keywords:** Gait, Machine learning, Older adults, Self-supervised learning, Accelerometer, Neurological disorders, Geriatrics, Biomedical engineering

## Abstract

Progressive gait impairment is common among aging adults. Remote phenotyping of gait during daily living has the potential to quantify gait alterations and evaluate the effects of interventions that may prevent disability in the aging population. Here, we developed ElderNet, a self-supervised learning model for gait detection from wrist-worn accelerometer data. Validation involved two diverse cohorts, including over 1000 participants without gait labels, as well as 83 participants with labeled data: older adults with Parkinson's disease, proximal femoral fracture, chronic obstructive pulmonary disease, congestive heart failure, and healthy adults. ElderNet presented high accuracy (96.43 ± 2.27), specificity (98.87 ± 2.15), recall (82.32 ± 11.37), precision (86.69 ± 17.61), and F1 score (82.92 ± 13.39). The suggested method yielded superior performance compared to two state-of-the-art gait detection algorithms, with improved accuracy and F1 score (p < 0.05). In an initial evaluation of construct validity, ElderNet identified differences in estimated daily walking durations across cohorts with different clinical characteristics, such as mobility disability (p < 0.001) and parkinsonism (p < 0.001). The proposed self-supervised method has the potential to serve as a valuable tool for remote phenotyping of gait function during daily living in aging adults, even among those with gait impairments.

## Introduction

Aging is associated with progressive loss of motor function. These deficits are heterogeneous and may manifest as reduced walking speed, poor balance, increased gait variability, increased fear of falling, and shorter stride length^1–3^. Objective measures of gait obtained during brief supervised gait testing in a lab or clinic predict survival, adverse health outcomes, and loss of independent living^4–6^. However, these brief assessments provide only a limited snapshot of an individual's gait abilities and may not fully reflect function and variability during the manifold demands of daily living^7,8^. Advances in unobtrusive sensor technology afford investigators the opportunity to obtain a more comprehensive assessment of mobility via remote multi-day recordings of daily living. However, the automated analytic tools employed for many commercially available devices focus nearly exclusively on healthy younger adults and do not account for the impairments observed in older adults during device development or validation^9,10^. Hence, there is an urgent need for the development and validation of automated tools to quantify daily living gait among the full health spectrum of older adults who reside in community-settings^11,12^.

Previous studies investigating real-world gait employed accelerometers worn on the lower back, leveraging the inherent quasi-periodicity of lumbar movement during walking^13^. While these studies have demonstrated the potential of assessing daily living gait, sensor placement on the lower back may present limitations for long-term adherence due to potential discomfort^14^. A different approach is to ask participants to wear a wrist-worn accelerometer. Wrist-worn accelerometers have gained widespread use to measure daily living physical activity ^15–18^. In this regard, the ubiquity and popularity of smartwatches make wrist-worn accelerometers a practical choice for ensuring high compliance in daily living studies. Wrist-worn accelerometers enable the extraction of a wide range of daily living behaviors, including sleep patterns^19^, circadian metrics^20^, and levels of physical activity^21^. While estimated physical activity levels can provide many insights^22,23^, to date, most studies using a wrist-worn accelerometer lacked detailed and high-resolution information about other crucial facets of gait quality^21^. Therefore, recent efforts have focused on leveraging these accelerometers to assess walking and gait quality.

The first step in deriving gait metrics from an accelerometer is the detection of gait sequences from the raw accelerometer signals^24,25^. Gait detection from a wrist-worn accelerometer is more challenging compared to other locations, such as lower limbs or lower back, due to the non-gait related hand movement and the fact that wrist movements often deviate from the expected periodic swinging during the gait cycle. This may occur, for instance, when an individual walks while simultaneously engaging in other activities, such as texting. This challenge is exacerbated for older adults and people with gait disturbances, such as Parkinson’s disease who manifest reduced arm swing during walking^26^. People with Parkinson’s disease also exhibit symptoms of tremor and dyskinesia, which impact wrist movements and contribute to an overall less stable and consistent gait pattern, factors complicating gait detection algorithms^24^.

Classical gait detection algorithms employ signal processing techniques, such as peak detection and wavelet analysis, to extract features both from the time and frequency domain^25,27^. These features are then used to identify gait sequences based on the repeated periodic waveforms manifested during gait. However, the complex wrist movements render the differentiation between gait and non-gait movements very challenging. Alternative approaches are needed to detect gait from wrist-worn accelerometers.

Previous studies addressed this goal by employing supervised machine learning algorithms that were trained to identify patterns in the signal associated with gait^16,28,29^. Kluge et al.^25^ conducted a comprehensive analysis of gait detection algorithms using accelerometer data from lower-back and wrist-worn accelerometers. The algorithms were trained on data from healthy young adults and subsequently tested on diverse subsets of adults from the Mobilise-D technical validation study^30^, including older adults with and without varied diagnoses. They found, not surprisingly, that algorithms based on lower-back data outperformed wrist-based algorithms. Yet, the reduced performance of wrist-based algorithms may be attributed, in part, to being trained on data from healthy young adults, potentially leading to suboptimal performance among older adults. This highlights the need to optimize wrist-based algorithms for older adults, who more commonly show heterogeneous gait abnormalities that do not occur as frequently in younger adults.

The best performing wrist-based algorithm identified in the study by Kluge et al. was initially developed and validated in Brand et al.^24^. In that study, we employed a supervised convolutional neural network with U-Net architecture^31^ for gait detection, focusing on older adults and people with Parkinson’s disease (PD). The results were then compared to those of a control group comprising healthy young adults. Our findings indicated that biological meaningful measures of gait quality (e.g., cadence and gait regularity) and quantity (e.g. daily walking duration) could be derived from a wrist-worn accelerometer. However, it is crucial to note that the model's performance was reduced when applied to older adults and individuals with PD, compared to the healthy young adult control group. An important impediment for training a supervised model that can be applied to older adults and varied clinical conditions derives from the scarcity of ground-truth labels indicating the temporal location of the gait sequences, especially for recordings of unsupervised movement during daily living.

Recently, there has been a growing interest in leveraging self-supervised learning (SSL) methods to overcome the gap imposed by the shortage of labeled data^32^. SSL generally comprises two main stages. First, learning feature representations of the signals using a substantial amount of unlabeled data, which can be achieved through methods such as multi-task learning (MTL)^33^ and contrastive learning^32,34^. An example of contrastive learning is the SimCLR method: “A Simple Framework for Contrastive Learning of Visual Representations”^32^. In these approaches, the model's objective is to predict characteristics of the signal that do not require any labels. This stage is commonly referred to as the 'pretext' stage. The second stage involves fine-tuning the SSL model with a smaller set of labeled data in a supervised manner for a downstream task (e.g., gait detection).

The SSL approach has demonstrated significant potential in several human activity recognition tasks^35–37^. For example, Yuan et al.^38^ utilized the UK Biobank dataset, which comprised daily living recordings from a wrist-worn accelerometer, to develop an SSL model for activity recognition and exhibited improved performance in several tasks and datasets. Small et al.^39^ fine-tuned this SSL model for gait detection on a semi-living dataset, termed OxWalk, which included approximately one hour of recording in a home environment. However, the dataset used for fine-tuning included only healthy adults (N = 39, mean age = 38.5 years). Thus, their model may not be optimized for older adults or individuals with gait disturbances.

Here, we developed and evaluated a gait detection deep learning approach, termed ElderNet, that was oriented and optimized for older adults and, in particular, those who might have impaired gait. The first stage involved the training of an SSL model, utilizing the pre-trained UK Biobank model of Yuan et al.^38^. This SSL model was extensively modified in both architecture and training cohorts to include a large unlabeled dataset of more than 1000 older adults with and without impaired gait who wore a wrist-worn device for up to 10 days (mean age 83 years old) and were participating in the RUSH Memory and Aging Project (MAP)^40–42^. Next, we fine-tuned the model on a labeled dataset consisting of 83 older adults (mean age = 71.9 years) from the Mobilise-D technical validation study^30^, each wearing a wrist-worn accelerometer for approximately 2.5 h. The Mobilise-D dataset is one of the largest available labeled datasets that include daily living recordings from a wrist-worn accelerometer in older adults. It contains a ground-truth reference for indicating the presence or absence of gait sequences. Additionally, the dataset contains participants with different health conditions, presenting a range of gait patterns, including individuals with Parkinson's disease, proximal femoral fracture, chronic obstructive pulmonary disease, congestive heart failure, and healthy adults. To explore the added value of the putative enhancements of the ElderNet, we compared it to two state-of-the-art algorithms: the U-Net architecture, which achieved the highest results in the study by Kluge et al.^25^, and the model developed by Small et al., termed OxWalk, utilizing the strong UK Biobank SSL model.

Finally, we applied ElderNet to a set of new participants—not previously trained by the model—to begin to explore its construct validity and generalizability. Construct validity refers to the degree to which a measurement tool, like ElderNet, accurately evaluates its intended purpose, specifically gait detection. In this context, we examined walking duration obtained through ElderNet across cohorts whose clinical status is likely to lead to reduced daily living walking.

## Results

### Performance of the gait detection algorithm

**Fig. 1:**
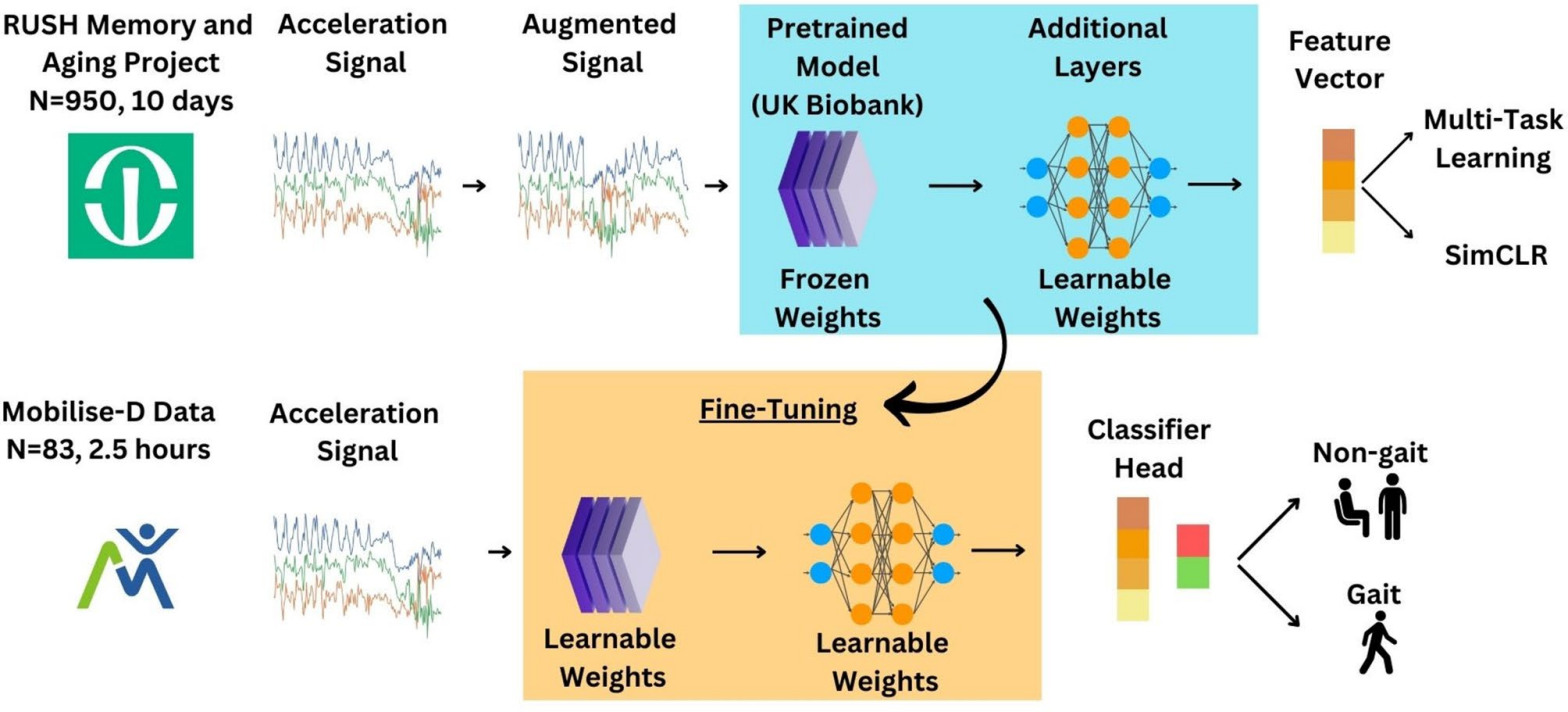
The ElderNet pipeline. In the SSL phase, data was segmented into non-overlapping 10-s windows. These windows underwent signal augmentations and were used as input for the SSL model, composed of the UK Biobank pre-trained model and additional de-novo optimized layers, producing a feature vector. In the MTL task, the feature vector undergoes a linear transformation to generate binary predictions of the multiple possible augmentations. In the SimCLR task, the loss is calculated directly from the feature vector. The weights from the SSL phase were then fine-tuned to train a supervised gait detection model using labeled data from the Mobilise-D dataset.

To develop ElderNet, an SSL model was trained using the MAP database constituting 950 participants. Next, the labeled data from Mobilise-D was used for fine-tuning ElderNet and evaluating its performance (Fig. 1). 83 participants were included in the Mobilise-D dataset. Table 1 summarizes the characteristics of the Mobilise-D dataset.

**Table 1:** Characteristics of older adults in the Mobilise-D technical validation study.

	HA	CHF	COPD	PD	PFF
No. of participants (N)	20	11	17	19	16
Age (years)	71.7 ± 5.8	69.1 ± 11.7	69.4 ± 9.1	69.3 ± 7.0	79.9 ± 8.2
Gender (M:F)	11:9	7:4	9:8	15:4	7:9
No. of participants with a walking aid (and percentage)	0	4 (36%)	0	1 (5%)	6 (38%)
Montreal cognitive assessment (0–30)	27.7 ± 2.6	27.1 ± 2.9	24.6 ± 3.3	24.7 ± 3.9	23.8 ± 4.3
CAT score (0–40)	N/A	19.65 (8.95)	N/A	N/A	N/A
FEV_1_ (L)	N/A	1.58 (0.58)	N/A	N/A	N/A
Hoehn and Yahr stage (N)	N/A	N/A	N/A	I: 4, II: 10, III: 5	N/A
SPPB (0–12)	N/A	N/A	N/A	N/A	5.94 (3.96)
Days since injury	N/A	N/A	N/A	N/A	132.1 (122)
No. of gait sequences per recording	66.5 ± 27.7	54.9 ± 31.9	60.8 ± 25.5	35.7 ± 23.6	34.0 ± 23.8
Average length of gait sequences (s)	30.7 ± 25.2	29.6 ± 18.0	17.6 ± 5.0	32.6 ± 22.0	29.1 ± 16.4
Gait percent (%) per recording	18.0 ± 9.3	16.3 ± 17.2	10.4 ± 3.5	14.3 ± 11.2	11.5 ± 9.4

*HA* healthy adults, *CHF* congestive heart failure, *COPD* chronic obstructive pulmonary disease, *PD* Parkinson’s disease, *PFF* proximal femoral fracture, *CAT* chronic obstructive pulmonary disease assessment test, *FEV*_1_ forced expiratory volume in 1 s, *SPPB* short physical performance battery, *SPPB* short physical performance batter; scores on this test range from 0 to 12 (best performance) and values less than 10 indicates one or more mobility limitations and lower SPPB scores have been associated with an increased risk of disability^43^.

**Fig. 2:**
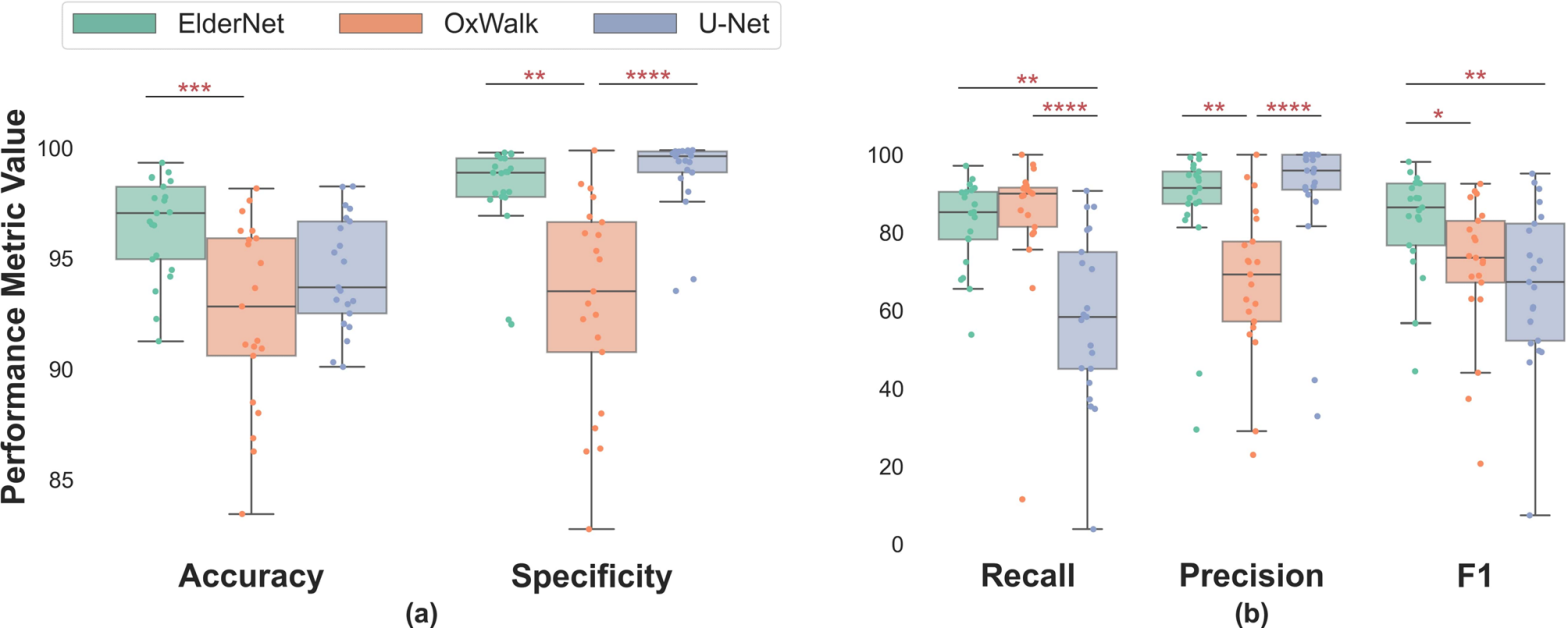
Comparing the proposed ElderNet approach with two state-of-the-art methods. Performance was calculated using the unseen Mobilise-D test set. For each model, individual points represent individual participants (N = 21). *p < 0.05; **p < 0.01; ***p < 0.001; ****p < 0.0001 (Bonferroni corrected).

**Fig. 3:**
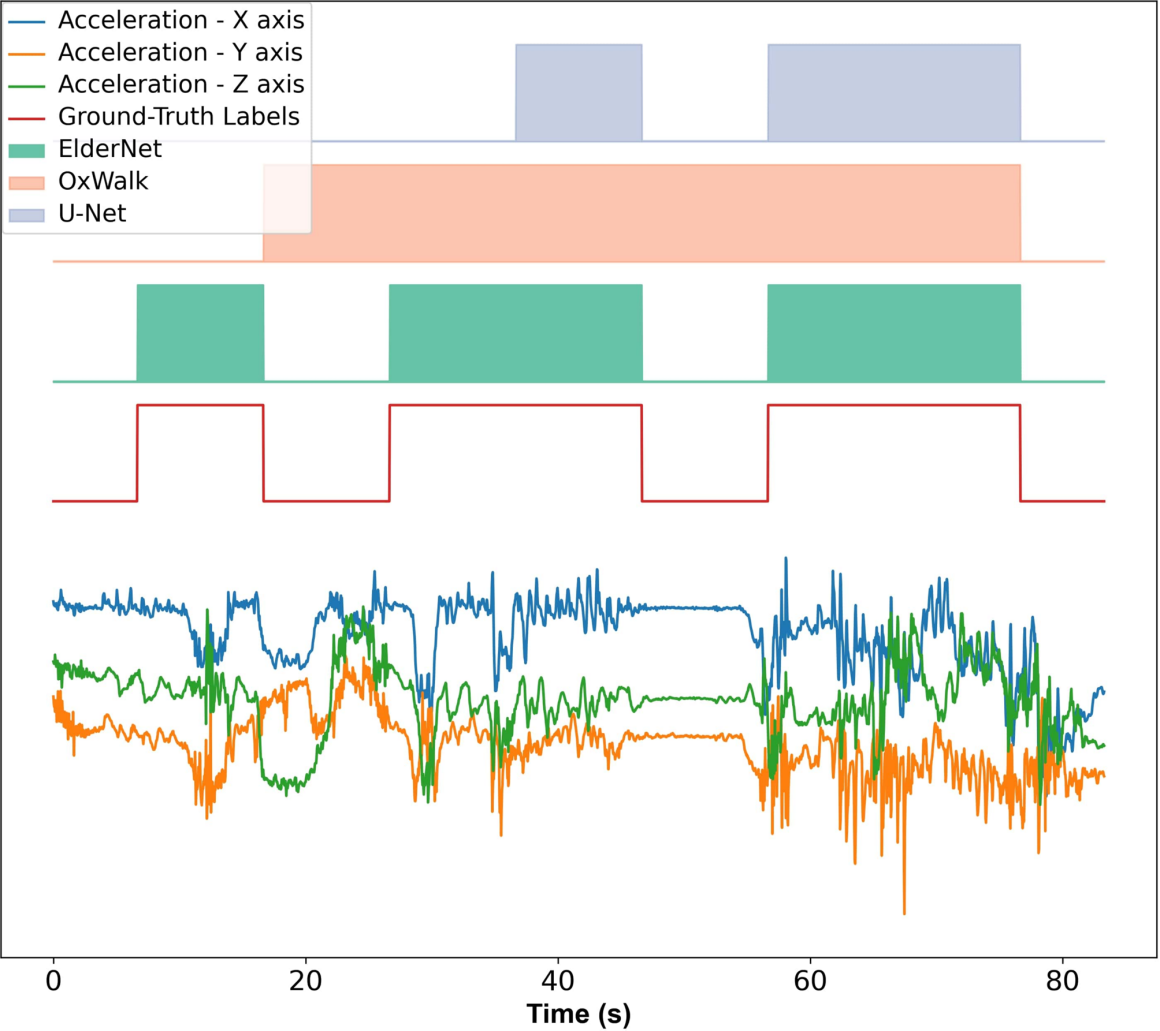
A representative real-world signal from the unseen Mobilise-D test set alongside the corresponding predictions from the proposed (ElderNet) and reference (OxWalk and U-Net) models. The lower signal displays raw acceleration data in three axes. The red rectangles indicate actual gait sequences based on ground-truth labels.

The model predictions made by ElderNet significantly outperformed the two other state-of-the-art algorithms^24,39^ both in terms of accuracy and F1 score. The median accuracy for ElderNet was 96.86%, surpassing the U-Net at 93.69%, and OxWalk at 92.83% (p < 0.001). In terms of F1 scores, ElderNet achieved a score of 86.52%, outperforming the U-Net and OxWalk models which achieved scores of 67.29% (p = 0.046) and 73.51% (p < 0.01), respectively (Fig. 2). ElderNet exhibits high results across all the various cohorts, with F1 scores above 80% for all cohorts (Table 2). While the other models tended to poorly identify the gait bouts for the PFF cohort (with average F1 score of 56.29% and 51.53% for the OxWalk and U-Net, respectively), ElderNet achieved higher F1 score of 81.95% for this cohort. Figure 3 shows a representative example of a raw acceleration signal containing gait sequences, along with the predictions of the different models and the corresponding ground-truth labels.

**Table 2:** Performance of the algorithms across various cohorts in the Mobilise-D test dataset.

	HA	CHF	COPD	PD	PFF
OxWalk	71.75 ± 16.65	72.41 ± 8.97	72.83 ± 4.51	79.9 ± 23.82	56.29 ± 24.08
U-Net	72.57 ± 13.69	69.1 ± 4.46	56.40 ± 9.39	82.75 ± 20.31	51.53 ± 31.58
ElderNet	82.82 ± 15.08	82.21 ± 4.82	84.17 ± 7.83	83.23 ± 22.34	81.95 ± 12.08

Performance is reported as the F1 score (± SD). 19 participants from the Mobilise-D used for test-set. Model performance was based on comparison with reference values (labels) obtained from the multi-sensor INDIP (INertial module with DIstance sensors and Pressure insoles) system.

*HA* healthy adults, *CHF* congestive heart failure, *COPD* chronic obstructive pulmonary disease, *PD* Parkinson’s disease, *PFF* proximal femoral fracture.

### Model selection: the effect of the SSL approach

We compared the performance of different SSL approaches, specifically MTL and SimCLR, with different model heads on top of the pre-trained UK Biobank model. The results showed that the fully-connected head with non-linearity performed the best under MTL, achieving the highest F1 score of 84.74 ± 0.51 (Table 3). This configuration was selected for further evaluations.

**Table 3:** The effect of the SSL approach.

Model's head	MTL	SimCLR
FC (without non-linearity)	84.67 (0.44)	84.52 (0.38)
FC (with non-linearity)	84.74 (0.51)	84.57 (0.76)
U-Net	83.02 (0.86)	84.28 (0.45)

Performance is reported as the F1 score (standard deviation between different seeds of validation, see the “Methods” section for more details).

*FC* fully-connected.

### The impact of self-supervised learning

We compared ElderNet with its supervised counterpart (Table 4). ElderNet exhibited superior performance compared to its supervised counterpart, achieving an F1 score of 84.74 for ElderNet, compared to 79.21 for the supervised model.

**Table 4:** The effect of using SSL compared to supervised counterparts.

Model's head	ElderNet	Supervised
FC (without non-linearity)	84.67 (0.44)	79.08 (0.24)
FC (with non-linearity)	84.74 (0.51)	79.21 (0.31)
U-Net	83.02 (0.86)	78.77 (0.73)

Performance is reported as the F1 score (standard deviation between different seeds). In this table, we employed the same architecture (ResNet-V2 + additional model’s head), once using the ElderNet configuration and once by performing fine-tuning from scratch without utilizing the UK Biobank pre-trained model and the MAP data.

*FC* fully-connected.

### Exploring construct validity

**Fig. 4:**
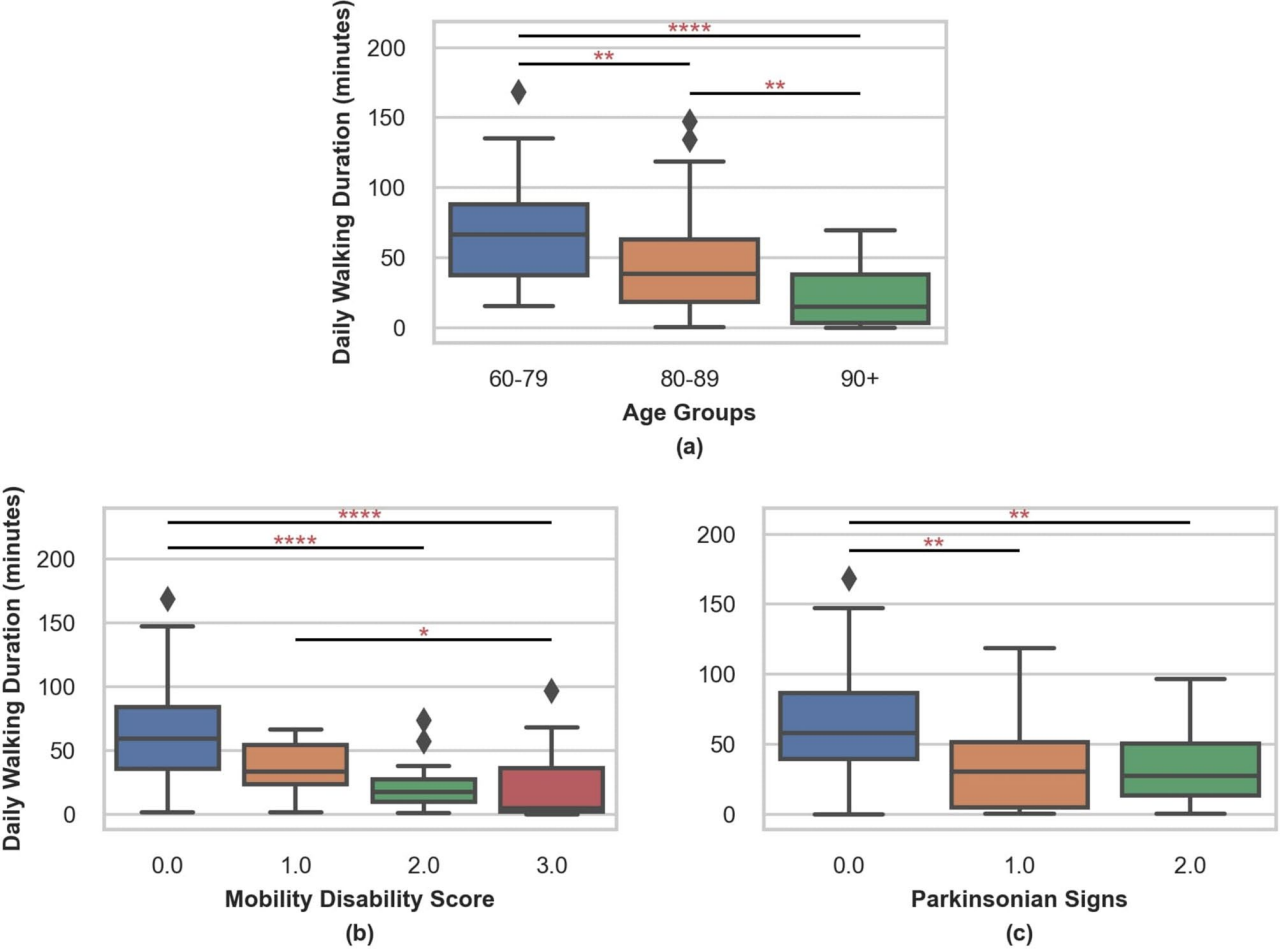
Distribution of daily walking duration across various groups: age, mobility disability scores (where 0 indicates no mobility disability), and the count of parkinsonian signs (with 0 indicating no signs, 1 denoting one sign, and 2 representing 2 or more signs). Differences between these groups were evaluated using the non-parametric Kruskal–Wallis test. The asterisks denote significant differences between cohorts, as determined by Dunn's post-hoc analysis. *p < 0.05; **p < 0.01; ***p < 0.001; ****p < 0.0001 (Bonferroni corrected). In partial correlation analyses that adjusted for age, sex, and BMI, walking duration remained significantly associated with mobility disability (rho = −0.37, p < 0.01) and with the number of parkinsonian signs (rho = −0.32, p < 0.01).

To examine the construct validity of the output of ElderNet, we first applied it on an unseen portion of the MAP dataset (N = 157) that was not utilized during the training of ElderNet. Table 5 summarizes the characteristics of this test dataset. A preliminary analysis based on the detected gait events, revealed a few statistically significant differences across different subject populations and disease cohorts. The average daily walking duration displayed variations among participants in different demographic and clinical groups, as demonstrated in Fig. 4. A significant difference in daily walking durations was observed between age groups, indicating a decline in walking activity with age, supporting its utility. To account for this, we performed partial correlation analyses, adjusting for age, sex, and BMI in subsequent comparisons, and found that the differences between groups remained statistically significant.

**Table 5:** Average daily walking durations across demographic and clinical factors in the MAP unseen dataset.

Characteristic	N (%)
Overall	157 (100)
Age (years)	
60–69	4 (2.55)
70–79	33 (21.02)
80–89	78 (49.68)
90–99	40 (25.48)
100 +	32 (1.27)
Sex	
Female	126 (80.25)
Male	31 (19.75)
BMI (kg/m^2^)	
Underweight (< 18.5)	5 (3.18)
Normal Weight (18.5–24.9)	46 (29.30)
Overweight (25–29.9)	60 (38.22)
Obese (≥ 30)	40 (25.48)
Unknown	6 (3.82)
Mobility disability score*	
0	74 (47.13)
1	28 (17.83)
2	28 (17.83)
3	24 (15.29)
Unknown	3 (1.92)
Falls in the past year	
0 falls	106 (67.52)
≥ 1 falls	49 (31.21)
Unknown	2 (1.27)
Parkinsonism	
No parkinsonism	37 (23.57)
Possible parkinsonism	35 (22.30)
Parkinsonism	39 (24.84)
Unknown	46 (29.30)

*0 indicates the absence of mobility disability. 3 indicates a high level of disability.

Furthermore, Fig. 4 illustrates that participants with a mobility disability score of 0 (no mobility disability) walked significantly more minutes per day than those with scores of 2 (p < 0.0001) and 3 (p < 0.0001). Additionally, participants with a mobility disability score of 1 also showed a significant difference from those with a score of 3 (p = 0.048). Examining participants with different parkinsonism scores in terms of the number of parkinsonian signs, we observed that individuals without any parkinsonian signs walked significantly more than those with 1 sign (p < 0.01) or two or more signs (p < 0.01).

## Discussion

In this work, we developed and validated a gait detection algorithm (ElderNet), specifically designed for older adults with and without gait impairments. ElderNet demonstrated superior performance compared to the two state-of-the-art algorithms. It achieved the highest accuracy, significantly surpassing the OxWalk model^39^. Moreover, its F1 score was higher than both OxWalk and the U-Net^24^ models. Additionally, ElderNet achieved at least comparable results in other metrics such as specificity, recall, and precision. The imbalance between gait and non-gait sequences in daily living is often expressed by a significant trade-off between precision and recall^24^. While the U-net and the OxWalk models indeed exhibited such a trade-off, our model was prominent with stable precision *and* recall, resulting in a high F1 score. This suggests that ElderNet is well-suited for daily living data, capable of identifying most existing gait sequences (i.e., high recall) with high confidence (i.e., high precision).

ElderNet exhibited stable and high performance across all different cohorts (recall Table 2). Importantly, it performed relatively well on the PFF cohort, patients with poorer mobility as indicated by low scores on the SPPB, where other state-of-the-art algorithms encountered difficulties. In this study, participants in the PFF cohort were, on average, 132.1 days (approximately19 weeks) post-surgery and had a mean SPPB score of 5.9. This aligns with prior studies that reported SPPB scores ranging from 4 to 8 approximately 12 weeks post-hip fracture^44,45^. Previous studies have reported lower performance in the PFF cohort, attributing it to several factors that significantly impact the accuracy of gait detection algorithm^25,46^. Patients with PFF often demonstrate altered gait patterns due to pain, muscle weakness, and impaired mobility. Additionally, their gait may be asymmetric, making it challenging for algorithms to identify regular gait patterns. Therefore, ElderNet's success in identifying gait sequences in this cohort highlights its potential to detecting gait even in individuals with relatively impaired gait.

Gait detection algorithms often lack labeled data from daily living datasets, particularly for older adults and individuals with gait impairments. This scarcity of labeled data prevents the algorithms from being optimized for these populations, whose gait signals can be diverse and abnormal. Here, an SSL method was utilized to address this gap. First, a pre-trained model trained on the UK Biobank data was leveraged. The UK Biobank dataset consists of 100,000 participants who wore a wrist-worn accelerometer in their daily lives, making it the largest dataset of its kind. Due to its size, we anticipated benefits from incorporating this pre-trained model into our SSL phase. Indeed, utilizing this pre-trained model led to a higher F1 score (82.59) than training the SSL model from scratch (F1 score of 77.15, Supplementary Table S1).

Our objective was to develop a gait detection algorithm tailored for older adults, aiming to bridge the current accuracy gap observed in algorithms designed for this population^24^. While the UK Biobank dataset included a large number of older adults, its participants were recruited in the age range of 45–69, with a mean age of 62 for the visits that involved wearing the wrist accelerometer. To address this limitation, we leveraged the MAP dataset with a mean age of 83.6 years old (range 62–103) and more than 1000 participants. We found that integrating the MAP data into our combined model enhanced its overall performance (Supplementary Table S2). This improvement may be attributed to the fact that the extensive MAP data used to train ElderNet better represented the characteristics of the target population i.e., older adults that were also reflected in the test set Mobilise-D data.

Two different SSL approaches were explored, namely MTL and SimCLR. Overall, both methods yielded similar performance, with a slight advantage favoring the MTL results, but with no significant difference (Table 3). These findings are consistent with a previously published paper that observed similar results for SimCLR and MTL in human activity recognition tasks using acceleration data from the wrist^35^. Finally, we compared ElderNet with its supervised counterpart (Table 4). Remarkably, our model exhibited superior performance compared to its supervised counterpart, underscores the potential of leveraging large unlabeled data to learn feature representations of the data.

In this study, the Mobilise-D data was utilized for the fine-tuning phase, leveraging its unique characteristics. Firstly, the dataset incorporates a robust reference system, the INDIP system, whose accuracy has been previously validated against an optical motion capture system. The results showed excellent absolute agreement (ICC > 0.95) within a laboratory setting^47,48^. Although the validation performance was lower in simulated daily activity tests, it was still relatively high, with an ICC > 0.86 for all cohorts except the PFF cohort, which had an ICC of 0.76. This establishes the INDIP system as a reliable method for obtaining reference data in real-world environments. Moreover, the Mobilise-D dataset contains daily living data from older adult populations, particularly those with specific medical conditions that affect mobility^25^. While we acknowledge that the 2.5-h assessment used in Mobilise-D data may not fully capture the complete variability of real-world walking, this dataset remains one of the largest available with comprehensive gait and non-gait reference information across various disease indications with labels.

Notably, the Mobilise-D cohort includes older adults who utilize walking aids, exhibiting abnormal gait signals from the wrist accelerometer, thereby complicating gait detection^25^. However, the test set in our study included only few participants who used walking aids during the recordings, limiting our ability to draw precise conclusions about gait detection stratified by walking aid usage. Future work should focus on exploring this aspect more deeply to understand the applicability of ElderNet in detecting gait patterns among older adults who use walking aids.

The establishment of ElderNet sets the stage for subsequent studies aimed at extracting meaningful digital mobility outcomes related to gait quantity and quality from the identified gait sequences^16,49^. Gait measures have already been shown to serve as potential biomarkers for age-related health outcomes^5,50^. Notably, gait speed has been shown to be associated with survival rates in older adults^51^. A recent study has demonstrated that using a simple model based solely on mean acceleration data can facilitate the prodromal diagnosis of Parkinson's disease^52^. We hypothesize that incorporating higher-level gait measures into such models can augment their predictive capabilities, leading to better identification of multiple neurological conditions that manifest with gait impairments.

It is important to highlight that we standardized the sampling rate of all datasets to 30 Hz to align with the frequency used in the pre-trained UK Biobank model. This relatively low sampling rate allowed for the efficient use of long-duration recordings. Exploring the ramifications of using different sampling rates should be addressed in future work. While the MAP data utilized in the SSL phase and the participants from the Mobilise-D data shared similarities in their emphasis on older adults, there were notable differences between them. Particularly, the average age of the MAP is higher (83.6 years) than that of the Mobilise-D data (71.9 years). Additionally, the Mobilise-D dataset predominantly includes participants with specific medical conditions, unlike the MAP data which is not exclusively focused on populations with diseases. We attempted to address this by standardizing both datasets (MAP and Mobilise-D) using a zero-mean unit-variance whitening^35^. However, we observed that standardizing the MAP data, but not the Mobilise-D data, resulted in improved outcomes (Supplementary Fig. S1).

The data was segmented into non-overlapping 10-s windows, both in the SSL and fine-tuning steps, to align with the UK Biobank pre-trained model, which utilizes the same window size. Consequently, we defined windows containing 5 s or more as gait windows in our labeled dataset, omitting gait sequences shorter than five seconds. However, this approach can lead to an underestimation of the number of gait sequences that occur in daily living. A potential consequence of this approach could be the estimated daily walking duration, as observed in the construct validity step (recall Fig. 4), which was found to be slightly lower than reported in the literature^53^. To address this issue, we explored the use of dense labeling, involving a shift to per-sample labels and outputs in the fine-tuning model. Despite this modification, the model's performance was found to be lower compared to using window-based labeling, and there was no meaningful change observed in the estimated daily walking time (Supplementary Table S3, Supplementary Fig. S2). This suggests that the alternative dense labeling strategy does not provide a significant improvement in capturing daily walking patterns.

We evaluated ElderNet's performance based on the length of gait sequences, specifically comparing sequences shorter than 30 s to those longer than 30 s (see supplementary Table S4). The results show that ElderNet performs better on longer sequences (> 30 s), particularly in terms of precision. For shorter sequences, the precision was 76.28%, whereas for long sequences, it was 100%, indicating no false positives for sequences longer than 30 s. These findings are consistent with previous studies that demonstrated higher gait detection performance in longer sequences^29^. This could be due to the higher stability of the acceleration signal within a window (10 s) during longer activities. Since many daily living gait bouts are short, future work should consider ways of improving gait detection performance for short walking bouts.

## Conclusions

This study introduced ElderNet, a novel gait detection model developed and validated for older adults with and without known health conditions that can affect gait. The model demonstrated high performance in accurately identifying real-world gait sequences extracted from wrist recordings. When applied to unlabeled daily living data, ElderNet successfully revealed differences between different clinical groups supporting further clinical testing of its efficacy. Given that many older adults experience gait impairments, a reliable system for gait quantification is crucial for obtaining a comprehensive characterization of gait function remotely during daily living. ElderNet addresses that need.

## Methods

This study was composed of four stages:

1. Self-supervised learning**:** training an SSL model on a large amount of unlabeled activity data to learn the feature representation of daily living acceleration data.
2. Fine-tuning: utilizing the model from the SSL step for training a supervised gait detection system (ElderNet) using labeled data.
3. Gait Detection Test Phase: comparing the results of the gait detection model with 2 state-of-the-art algorithms on an independent test set.
4. Exploring construct validity**:** applying ElderNet on another unseen dataset to examine the potential of gait-based analysis for identifying differences between cohorts of different clinical characteristics.

### Preprocessing

To maintain uniformity in comparison with state-of-the-art algorithms, we standardized the acceleration data across the various cohorts by resampling to a 30 Hz resolution and dividing the signals into 10-s non-overlap windows, following a methodology similar to the UK Biobank study^38,39^. We considered the window as a gait window only when half or more of it was labeled as gait. Given that the typical gait frequency is less than 10 Hz, the 30 Hz sampling rate surpasses the Nyquist frequency, preventing any loss of essential signal information.

### Stage 1: self-supervised learning

#### Participants and wearable sensors

Participants were community-dwelling older adults enrolled in an ongoing cohort study of chronic conditions of aging, known as Rush Memory and Aging Project^40–42^. A total of 1117 participants aged between 61 and 103 years (mean 83.77 ± 7.37 SD) (76% female) participated in the study. The dataset was divided into two sets: 85% of the data (N = 950, mean age = 83.6 ± 7.3 years, 76% female) was utilized for the SSL model training, while the remaining 15% (N = 167, mean age = 84.2 ± 7.6 years, 80% female) was reserved for construct validity step (see the construct validity section). Written informed consent was obtained, and the study was conducted by the latest version of the Declaration of Helsinki and was approved by Rush University Medical Center Institutional Review Board.

Participants wore the GENEActiv device (Activinsights Ltd.; Cambridgeshire, UK), a triaxial accelerometer, on their non-dominant wrist for 24 h/day for up to ten consecutive days. Acceleration data were sampled at either 40 Hz or 60 Hz, depending on the time of recording. Specifically, data recorded in the first half of 2018 were sampled at 60 Hz, while data recorded from the second half of 2018 onwards were sampled at 40 Hz. The device had a range of ± 8 gravitational acceleration units (g).

#### Self-supervised approaches

Typically, SSL models consist of a main trunk, usually a convolutional neural network, referred to as a feature extractor, which produces a vector containing feature representations. The feature vector is then adjusted to a different dimension to match the ‘pretext’ task associated with the chosen SSL approach. In this study, we investigated two SSL approaches, namely MTL and contrastive learning (SimCLR). We selected these approaches based on their demonstrated superior performance in downstream human activity recognition tasks, as identified through an extensive exploration of various SSL approaches using wearable sensors^35^.

In the MTL approach, each acceleration window undergoes data augmentation, where the objective of the model is to predict the augmentation of the signal (pretext task). Following the methodology of Yuan et al.^38^, 4 distinct augmentations were employed: (1) Reversing the signal. (2) Permutation of different segments of the window, with each segment comprising 10 samples. (3) Time warping, which alters arbitrary segments of the signal by stretching and compressing them. (4) Scaling each of the acceleration axes with a random factor. Each window has a random probability of undergoing each of the augmentations, and the model predicts whether the window underwent the augmentation, resulting in four binary outputs. The model’s loss is calculated using the cross-entropy function for all four augmentations and then averaged to produce the final loss.

The SimCLR contrastive learning method also employs data augmentations. In SimCLR, each window undergoes two augmentations, resulting in two distinct views of the same window. Views originating from the same source window are considered “positive” pairs, while views stemming from different sources are considered “negative” pairs. For instance, if we initially have N windows of acceleration signal, the transformation yields 2N views of the windows. Thus, for every positive pair of windows, there are 2N-2 negatives. In this study, we utilized a 3D rotation transformation as the augmentation function. In this augmentation, a random axis in 3D and a random rotation angle are drawn from a uniform distribution, and the corresponding rotation is applied to the window. This can be considered as a way to simulate different sensor placements^34^, making it especially effective for wrist accelerometers where the axis orientation frequently changes. We specifically chose this augmentation due to its demonstrated superior performance in downstream human activity recognition tasks associated with the SimCLR approach^34^. The different views of the windows pass through the model encoder (i.e., feature extractor), resulting in an output that reflects the different windows as feature vectors. Next, a contrastive loss function is employed to calculate the relationships between pairs of vectors using cosine similarity. The objective of the loss function is to maintain proximity in the feature space for vector representations of "positive pairs" while ensuring that "negative" pairs remain distant in this space. This loss is also known as the normalized temperature-scaled cross-entropy loss (NT-Xent)^32^.

#### Model configurations

To enhance the model's performance, the incorporation of a pre-trained model as the feature extractor of the SSL model was used. Specifically, we employed a model developed by Yuan et al.^38^, which utilized the diverse UK Biobank dataset to train an SSL model using the MTL approach. The architecture of the pre-trained model was ResNet-V2 with 18 layers. The input acceleration data underwent through the pre-trained model, resulting in an intermediate output- a vector with dimensions (1024, 1). Subsequently, we introduced additional layers on top of the pre-trained model, referred to as a model's head. The intermediate vector then traversed through these additional layers to produce the final output, suitable for the pretext task. While the weights of the pre-trained model were frozen during the training of our model, indicating they were not updated during gradient calculations, the weights of the model’s head were updated. This modification to the pre-trained model allowed us to tailor our model to older adults using the MAP data, considering that the pre-trained UK Biobank model did not exclusively focus on older adults. We termed our combined model ElderNet. Figure 1 illustrates the pipeline of our model.

We experimented with three different versions for the model's head, each with increasing complexity: (1) Using three fully-connected layers without non-linearity between them. (2) Using the same fully-connected layers, but with ReLU non-linear activation function between the layers. (3) Utilizing the U-Net with an architecture similar to the model employed during the testing phase. Supplementary Table S5 provides more details on the models' hyperparameters and implementation.

### Stage 2: fine-tuning

#### Participants and wearable sensors

For optimizing and evaluating algorithms for gait detection, a dataset from the Mobilise-D technical validation study was used. This multi-center observational dataset, originally aimed at validating real-world digital mobility outcomes included different patient and healthy populations. Participants were recruited at five sites: The Newcastle upon Tyne Hospitals NHS Foundation Trust, UK (Sponsor of the study) and Sheffield Teaching Hospitals NHS Foundation Trust, UK (ethics approval granted by London-Bloomsbury Research Ethics committee, 19/LO/1507); Tel Aviv Sourasky Medical Center, Israel (ethics approval granted by the Helsinki Committee, Tel Aviv Sourasky Medical Center, Tel Aviv, Israel, 0551-19TLV), Robert Bosch Foundation for Medical Research, Germany (ethics approval granted by the ethical committee of the medical faculty of The University of Tübingen, 647/2019BO2), University of Kiel, Germany (ethics approval granted by the ethical committee of the medical faculty of Kiel University, D438/18). Informed consent was provided by all participants to take part in the study and all research was performed in accordance with the Declaration of Helsinki. A comprehensive description of the study's experimental protocol, incorporating all inclusion and exclusion criteria, can be found in^30^.

Briefly, 112 participants across five different disease cohorts and one cohort of healthy adults were studied. The patient groups included chronic obstructive pulmonary disease, Parkinson's disease, multiple sclerosis, proximal femoral fracture, and congestive heart failure patients. We excluded the multiple sclerosis group (N = 20, mean age = 48.7 years) as we aimed to customize the model to older adults and the MS cohort comprises also young adults. In addition, nine participants were also excluded due to missing data, resulting in 83 participants overall used for this step. All participants gave written informed consent before participation. The participants were monitored during 2.5 h of real-world living undergoing their normal daily activities without a specific protocol. The participants were equipped with an accelerometer worn at the wrist on the non-dominant hand and a validated multi-sensor system, the INDIP as reference^30,47^. The INDIP system provided annotations (i.e., labels) regarding the temporal locations of the gait sequences.

#### Fine-tuning procedure

The fine-tuning step involved a supervised learning procedure. The model's input comprised the Mobilise-D dataset, which contains labels indicating the temporal location of the gait sequences. We divided the Mobilise-D data into 75–25%, where 75% of the data was used for training and validation of the supervised model, as well as for assessing different model configurations, and the remaining 25% was reserved for testing the model. We selected this ratio to ensure comparable distributions between the training and test sets, ensuring that each cohort has at least three participants in the test set. The divisions were made subject-wise, ensuring that the data points belonging to a particular subject were entirely contained within one subdivision and did not get shared across other subdivisions. We utilized the trained model from the SSL step to train a gait detection model. That is, the weights learned on the extensive unlabeled data served as a robust starting point for training a supervised gait detection model. To adapt the SSL model for gait detection, we modified its last layer to function as a linear layer producing a binary output (i.e., gait/non-gait). During the fine-tuning process, we allowed the model to update all of its weights. This decision was based on prior studies that demonstrated the preference for not freezing weights in the fine-tuning procedure^38,54^.

**Fig. 5:**
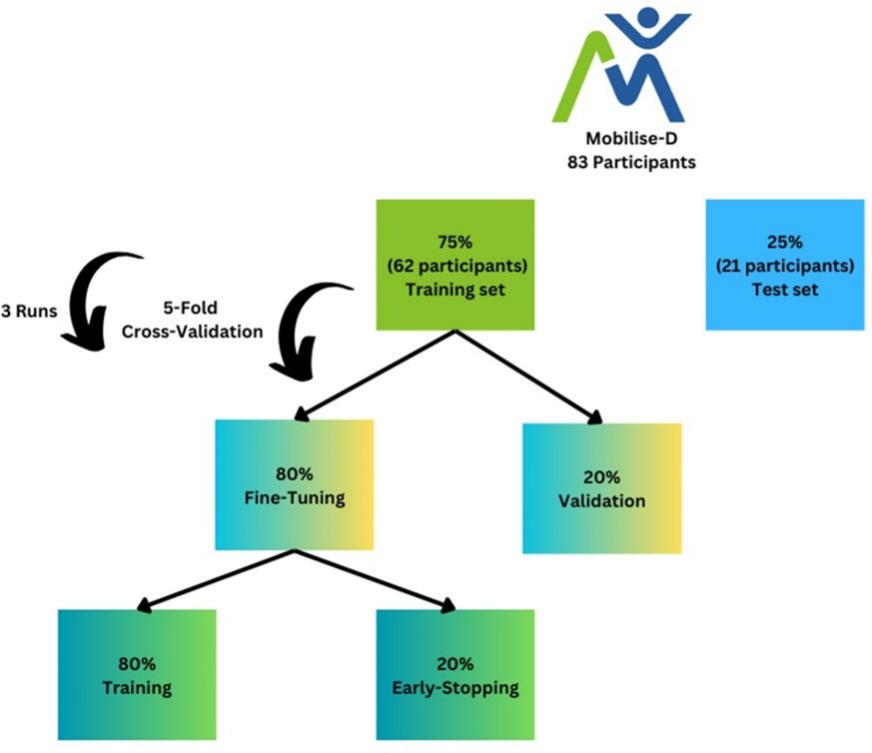
Mobilise-D dataset flow. Initially, 75% of the participants were utilized to fine-tune the model for the development of a gait detection model. A fivefold cross-validation approach was employed. During each fold, data was divided into 80% for training and 20% for validation. The model underwent training for 30 epochs, incorporating an early-stop mechanism that halted the training process if the loss on the set did not decrease for 5 consecutive epochs. Model performance evaluation was conducted on the validation set, and the final performance was calculated as the average across the 5 folds. This entire process was repeated three times for three different divisions of the cross-validation folds. The average of these three runs was then calculated. This approach allowed us to evaluate various model configurations and select the most effective one. Ultimately, the best configuration was applied to the 25% test set, comprising 21 participants, and compared against state-of-the-art models.

In the fine-tuning process, we again split the training set, corresponding to 75% of the entire data, into an 80–20 ratio. Eighty percent of this subset was used for training and 20% for validation. We applied five-fold cross-validation on the training set, stratified by class label and grouped by participant. An early-stopping mechanism was implemented to halt training when the loss stopped decreasing for five consecutive epochs. The cross-validation process was repeated with three different seeds, representing three different divisions of the folds, to obtain more generalizable results independent of a specific order of the data. The results from the three iterations were averaged to derive final performance metrics. The fine-tuning and performance evaluation processes were implemented for all different SSL configurations (refer to the Models Configuration section), utilizing only the training set. For each unique configuration, its performance after fine-tuning the Mobilise-D data was recorded. The configuration that yielded the best results was then selected as our model for comparison and further analysis. Figure 5 illustrates the flow of this process.

#### Ablation studies

To further explore the influence of different components of the SSL model on its downstream performance, several ablation studies were conducted. Initially, the impact of utilizing the pre-trained weights from the UK Biobank model was investigated^38^. For this purpose, the same architecture of the pre-trained SSL model was evaluated (i.e., ResNet) twice- once with the pre-trained weights from the UK Biobank model initialized, and once trained from random initialization on the MAP dataset.

To assess the contribution of the MAP dataset in tailoring the model to older adults, the combined network (the pre-trained model with the newly added layers) was utilized, and its performance was evaluated with and without utilizing the MAP data. This investigation allowed us to discern whether the performance difference stemmed solely from the expansion of the pre-trained model architecture (by adding the new layers) or if the use of a dataset focused on older adults, such as the MAP data, also played a role.

### Stage 3: testing

The F1 score from the fine-tuning step was used to select the best model configuration. The choice of using the F1 score for model selection is based on the inherent imbalance of daily living data in terms of gait, where gait sequences are much less frequent than non-gait ones. In imbalanced datasets, the F1 score provides a more realistic and unbiased assessment of the model's performance^24^**.** Model Performance was tested at the window level (i.e., comparing the prediction and the label of each window).

#### Model comparison

We compared the resulting ElderNet model with two state-of-the-art gait detection algorithms. The first comparison algorithm employed a U-Net architecture, developed and validated in our recent publication^24,25^. The U-Net model was originally trained on healthy young adults. The second model in our comparative analysis was an SSL algorithm pre-trained on the UK Biobank dataset and subsequently fine-tuned for gait detection in healthy adults, which was referred to as the OxWalk dataset^39^. We tested these 3 models on 25% of the Mobilise-D data, which was not used in the fine-tuning step. The performance metrics were calculated for each of the 21 participants in the test set, and then averaged to obtain the final performance.

### Stage 4: assessing construct validity

As a preliminary exploration of the clinical potential of the gait-detection information introduced by ElderNet, we applied the model to an unseen portion of the MAP dataset, ensuring that participants used in this step were distinct from those involved in the SSL phase. A total of 167 participants were assigned to this stage. To accurately analyze participant activity, we excluded time segments indicating participants who were not wearing the device. These non-wear periods were defined as consistent low movement (low STD) across all acceleration axes for at least 30 minutes^55,56^. For each participant, we extracted data from four full (24-h-long) days, as a recent study has shown that this duration provides reliable gait quantity measures^57^. Ten participants were excluded, due to an insufficient amount of activity (less than 96 h of data), resulting in a final number of 157 participants who were included in this stage. ElderNet was applied to the four days of data to identify the gait sequences. Subsequently, for each day, we summed the number of gait sequences and defined the median value as the daily walking time.

The Mobilise-D test set was not included for the construct validity investigation due to the relatively small sample size within each clinical cohort (only 3–5 participants per cohort). This small sample size would make it difficult to reliably explore associations between gait duration and disease severity. Using the training set (~ 75% of the Mobilise-D dataset) for construct validity is also not appropriate because the model was directly trained on this data for gait detection. Therefore, we used the larger and unseen MAP dataset for investigating construct validity.

To examine the construct validity of ElderNet, differences in daily walking time among participants belonging to different clinical cohorts were investigated. Specifically, we examined 2 motor-related clinical variables: the mobility disability score, assessed using the Rosow-Breslau scale^58^, and the number of parkinsonism signs^59^. The modified version of the motor portion of the United Parkinson's Disease Rating Scale (UPDRS III) was used to assess the presence of four Parkinsonian signs: bradykinesia, gait, rigidity, and tremor^60^. Participants were categorized into three cohorts (no sign, 1 sign, 2+ signs). We hypothesized that daily walking time would differ between these cohorts, with individuals without mobility disability spending more time walking than those with mobility disabilities^23^. Additionally, we expected individuals without Parkinsonian signs to spend more time walking than those with 1 or more Parkinsonian signs^61^.

### Statistical analysis

The Kruskal–Wallis test was performed to identify significant differences between ElderNet and state-of-the-art algorithms across the test performance metrics. Dunn's post-hoc analysis was applied to reveal the sources of difference among the models. In the context of construct validity, the Kruskal–Wallis test assessed differences in daily walking durations across cohorts with distinct demographic and clinical statuses. The corresponding Dunn's post-hoc analysis was then used to pinpoint the sources of variation in walking durations. To address multiple comparisons in all instances, the Bonferroni correction was applied. The Kruskal–Wallis test and Dunn's post-hoc analysis were implemented using the 'kruskal' function from the scipy.stats library and the posthoc_dunn function from the scikit_posthocs library, respectively. Partial correlation analyses were performed to adjust for age, sex, and BMI, using IBM SPSS Statistics software (Version 29.0.0.0).

## Supplementary Information

Supplementary Information.

## Data Availability

Raw data of a representative participant (dataset YAR, participant 0002) can be found on Zenodo: https://doi.org/10.5281/zenodo.7185429. The full data set will be made available by the Mobilise-D consortium after June 2024. All MAP data included in these analyses are available via the Rush Alzheimer’s Disease Center Research Resource Sharing Hub, which can be found at www.radc.rush.edu (accessed on 17 April 2023). It has descriptions of the studies and available data. Any qualified investigator can create an account and submit requests for deidentified data.

## Code availability

The code supporting this study is accessible on GitHub at the following link: https://github.com/yonbrand/ElderNet.
