# Supplementary material for: Self-supervised learning of wrist-worn daily living accelerometer data improves the automated detection of gait in older adults
Source: Sci Rep. 2024 Sep 6;14:20854. doi: 10.1038/s41598-024-71491-3 (PMC11379690; doi:10.1038/s41598-024-71491-3)
Supplement: Supplementary file 1 — Supplementary Information. [file 41598_2024_71491_MOESM1_ESM.pdf]

Self-Supervised Learning of Wrist-Worn Daily Living Accelerometer Data Improves the Automated Detection of Gait in Older Adults

Yonatan E. Brand (1,2), Felix Kluge (3), Luca Palmerini (4,5), Anisoara Paraschiv-Ionescu (6), Clemens Becker (7, 8), Andrea Cereatti (9), Walter Maetzler (10), Basil Sharrack (11), Beatrix Vereijken (12), Alison J. Yarnall (13,14,15), Lynn Rochester (13,14,15), Silvia Del Din (13, 15), Arne Muller (3), Aron S. Buchman (16), Jeffrey M. Hausdorff (2,16,17,18), Or Perlman (1,18)*

1 Department of Biomedical Engineering, Tel Aviv University, Tel Aviv, Israel

2 Center for the Study of Movement, Cognition and Mobility, Neurological Institute, Tel Aviv Sourasky Medical Center, Tel Aviv, Israel

3 Biomedical Research, Novartis Pharma AG, Basel, Switzerland

4 Department of Electrical, Electronic and Information Engineering Guglielmo Marconi, University of Bologna, Bologna, Italy

5 Health Sciences and Technologies - Interdepartmental Center for Industrial Research (CIRI-

SDV), University of Bologna, Bologna, Italy

6 Laboratory of Movement Analysis and Measurement, Ecole Polytechnique Federale de Lausanne, Lausanne, Switzerland

7 Robert Bosch Gesellschaft für Medizinische Forschung, Stuttgart, Germany

8 Unit Digitale Geriatrie, Universitätsklinikum Heidelberg, Heidelberg, Germany

9 Department of Electronics and Telecommunications, Politecnico di Torino, Turin, Italy

10 Department of Neurology, University Medical Center Schleswig-Holstein Campus Kiel, Kiel, Germany

11 Department of Neuroscience and Sheffield NIHR Translational Neuroscience BRC,

Sheffield Teaching Hospitals NHS Foundation Trust, Sheffield, UK

12 Department of Neuromedicine and Movement Science, Norwegian University of Science

and Technology, Trondheim, Norway

13 Translational and Clinical Research Institute, Faculty of Medical Sciences, Newcastle University, Newcastle upon Tyne, UK

14 The Newcastle Upon Tyne Hospitals NHS Foundation Trust, Newcastle upon Tyne, UK

15 National Institute for Health and Care Research (NIHR) Newcastle Biomedical Research Centre (BRC), Newcastle University and The Newcastle upon Tyne Hospitals NHS Foundation Trust, Newcastle upon Tyne, UK

16 Rush Alzheimer’s Disease Center and Department of Orthopaedic Surgery, Rush University Medical Center, Chicago, IL, USA

17 Department of Physical Therapy, Faculty of Medical & Health Sciences, Tel Aviv University, Tel Aviv, Israel

18 Sagol School of Neuroscience, Tel Aviv University, Tel Aviv, Israel

*Correspondence: Or Perlman, Email: orperlman@tauex.tau.ac.il

Supplementary Information

Ablation Studies

The supplementary tables present evaluations of various model configurations aimed at optimizing ElderNet, reporting performance using the F1 score and standard deviation across three seeds. Given the limited number of seeds, p-values were not reported as they would not provide meaningful statistical insights.

Supplementary Table S1. The effect of using the UK Biobank pre-trained model.

Model	Trained from scratch	Pre-trained model
ResNet-V2	77.15 (0.30)	82.59 (0.89)

Performance is reported as the F1 score (standard deviation between different seeds). The pre-trained UK Biobank model was a ResNet-V2 with the MTL approach. We employed the same architecture and MTL approach to train the SSL model from scratch using the MAP data.

Supplementary Table S2. The effect of customizing the SSL model for older adults using the MAP data.

Model's head	MAP	Without MAP
FC (without non-linearity)	84.67 (0.44)	82.55 (0.08)
FC (with non-linearity)	84.74 (0.51)	83.21 (0.45)
U-Net	83.02 (0.86)	83.90 (0.08)

Performance is reported as the F1 score (standard deviation between different seeds). In this table, we employed the combined model (i.e., pretrained UK Biobank model + additional model's head) with the optimal SSL configuration, termed ElderNet. FC: Fully-connected.

Supplementary Table S3. The effect of dense labeling.

Model	Window Labels	Dense Labeling
ElderNet	84.74 (0.51)	81.99 (0.41)

Performance reported as the F1 score (standard deviation between different seeds). The model employed for both labeling approaches is identical. The key distinction lies in the final

layer of the model: in the first approach, it projects the output for each window, while in the dense labeling approach, it projects the output for each sample.

Supplementary Table S4. Performance stratified by sequence length.

Sequence length	Accuracy	Specificity	Recall	Precision	F1 score
<30 seconds	95.99	97.54	80.29	76.28	78.23
>30 seconds	99.20	100.00	81.25	100.00	89.66

Supplementary Table S5. ElderNet setting.

Method	Optimizer	Learning-rate	Batch size	Epochs	Learning-rate Scheduler
MTL	Adam	1e-4	6000	40	Linear scaling with a 5-epoch warm-up
SimCLR	Adam	1e-4	6000	40	Cosine Decay with a 5-epoch warm-up

Each batch contains 1500 windows from 4 unique participants. The windows were sampled in proportion to their STD, as described in Yuan et al.[38]. For the MTL, we employed linear scaling for the learning rate to align with the pre-trained UK Biobank model, which utilized MTL and linear scaling. For SimCLR, we utilized the cosine decay scheduler, as outlined in the original paper of this method[32].

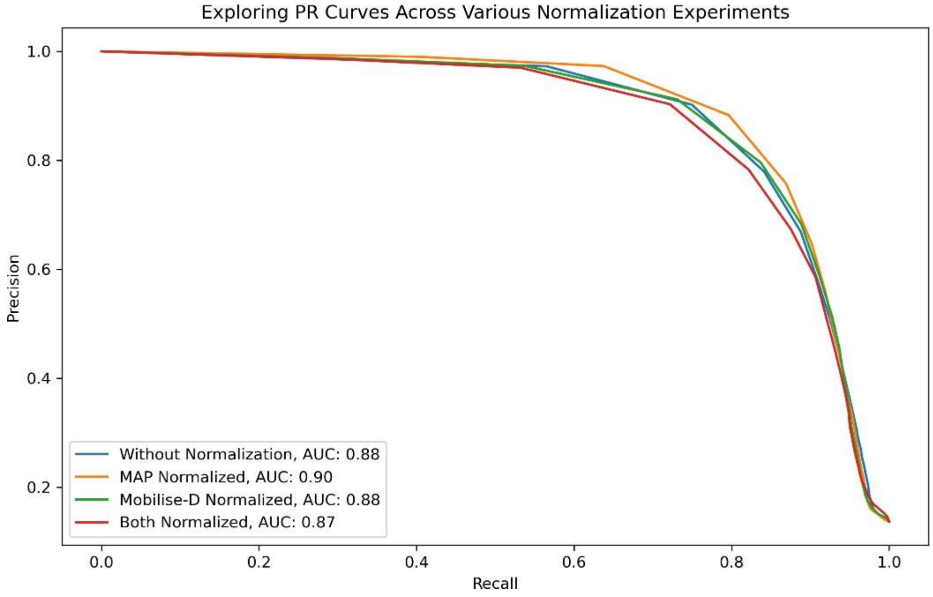

Supplementary Figure S1. Precision-Recall curves for different configurations of normalizations. Normalization was implemented per subject, making each axis of its acceleration signal zero-mean and a standard deviation of 1. AUC: Area under the curve.

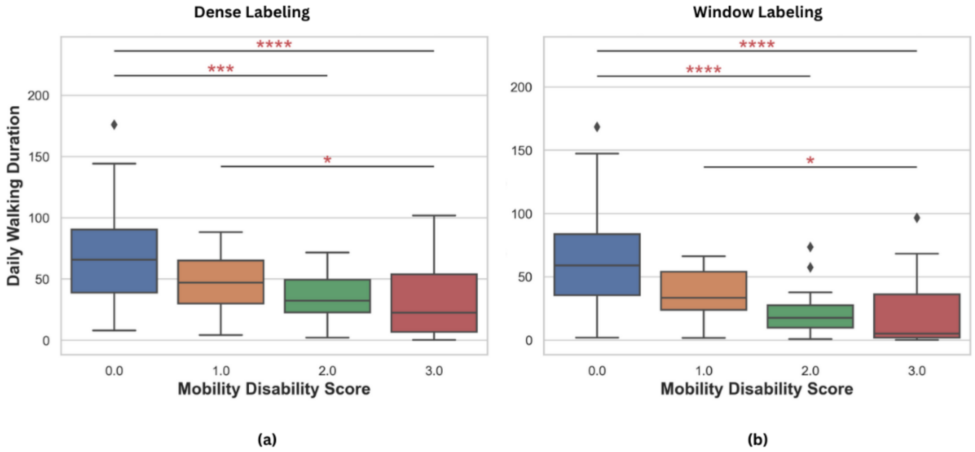

Supplementary Figure S2. a) On the left, it illustrates the estimated daily walking durations using a model that utilized dense labeling in the fine-tuning phase and provided a per-sample output. b) On the right, the figure represents the estimated walking duration using a model that outputs per-window prediction
